# Development and Application of Desorption Electrospray Ionization Mass Spectrometry for Historical Dye Analysis

**DOI:** 10.1021/acs.analchem.2c03281

**Published:** 2023-03-01

**Authors:** Edith Sandström, Chiara Vettorazzo, C. Logan Mackay, Lore G. Troalen, Alison N. Hulme

**Affiliations:** † EaStCHEM School of Chemistry, University of Edinburgh, David Brewster Road, Edinburgh EH9 3FJ, UK; ‡ National Museums Scotland, Collections Services Department, National Museums Collection Centre, 242 West Granton Road, Edinburgh EH5 1JA, UK

## Abstract

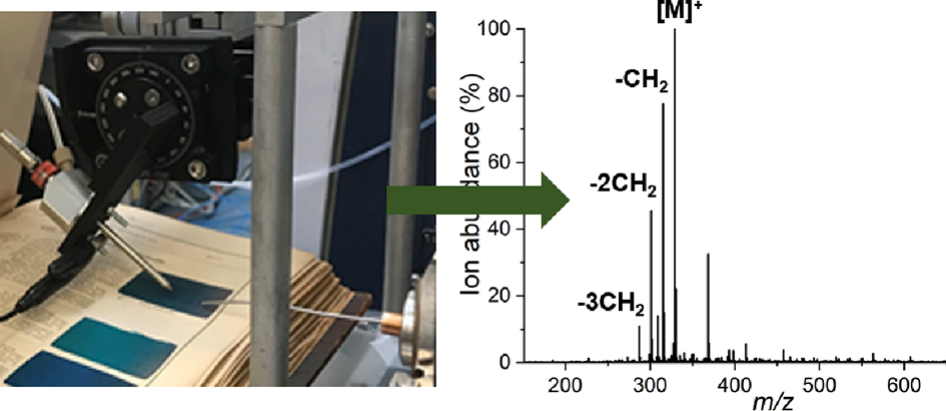

A desorption electrospray ionization (DESI) source was built and attached to a Bruker 7T SolariX FT-ICR-MS for the *in situ* analysis of 14 early synthetic dyestuffs. Optimization using silk and wool cloths dyed with rhodamine B concluded that when using a commercial electrospray emitter (part number: 0601815, Bruker Daltonik), a nebulizing gas (N_2_) pressure of 3.9 bar and a sprayer voltage of 4.5 kV (positive ionization mode) or 4.2 kV (negative ionization mode), a solvent system of 3:1 v/v ACN:H_2_O, and a sprayer incident angle, α, of 35° gave the highest signal-to-noise ratios on both silk and wool for the samples investigated. The system was applied to modern early synthetic dye references on silk and wool as well as historical samples from the 1893 edition of Adolf Lehne’s *Tabellarische Übersicht über die künstliche organischen Farbstoffe und ihre Anwendung in Färberei und Zeugdruck* [*Tabular overview of the synthetic organic dyestuffs and their use in dyeing and printing*]. The successful analysis of six chemically different dye families in both negative and positive modes showed the presence of known degradation products and byproducts arising from the original synthetic processes in the historical samples. This study demonstrates the applicability and potential of DESI-MS to the field of historical dye analysis.

In 1856, young William H. Perkin accidentally discovered the first synthetic dye mauveine and revolutionized both the chemical industry and textile manufacturing field. Other coal-tar-derived synthetic dyes quickly followed, and the superior range of colors, increased lightfastness, and low cost of the early synthetic dyes meant that they rapidly gained popularity over natural dyes. Although natural dyes are still used today, most industrial dyes have a synthetic origin.^1^ The study of early synthetic dyes in historical textiles is therefore not only important from a conservation and display perspective but it also aids the understanding of a very transitional and dynamic period in history. However, the low concentrations of dyestuffs and complex mixtures often present in historical textiles as well as dye and cloth degradation make dye analysis a challenging part of heritage science. Hence, a variety of invasive and noninvasive techniques are in use in the field. Here, an invasive technique is defined as a method requiring a sample being physically removed from the object and a noninvasive technique one that is used on samples *in situ*. A destructive technique consumes matter, while a nondestructive technique has no physical impact on the sample. A technique can therefore be noninvasive but destructive. The main analytical techniques in dye analysis are (ultra)high-performance liquid chromatography ((U)HPLC) and mass spectrometry (MS) due to their high sensitivity and selectivity;^2−6^ both these techniques are invasive and destructive. Although several protocols have been developed that only require small samples,^7^ the necessity to sample historical objects is a limitation often neither desirable nor possible. Noninvasive and nondestructive methods have been developed to circumvent this issue, such as fiber-optic reflectance spectroscopy^8,9^ and hyperspectral imaging,^10^ but these methods require significant modeling for data interpretation due to the complexity of dyestuff mixtures often found in historical objects.

The development of noninvasive ambient ionization techniques, such as desorption electrospray ionization (DESI),^11^ matrix-assisted laser desorption electrospray ionization (MALDESI),^12^ and direct analysis in real time (DART),^13^ at the start of the millennium offers an exciting opportunity to gain a similar level of information as invasive analyses using a noninvasive and micro-destructive technique, albeit lacking the separation of components achieved by chromatographic workflows. Textile fibers have been studied using ambient MS but mostly in a forensic or environmental context.^14,15^ Although DESI-MS has been attempted without success for dye analysis^16^ and a DESI source was recently developed for the study of ink in manuscripts,^17^ only DART-MS has been used for the study of dyes in historical textiles with success.^18−21^ In this study, a DESI source has been built, optimized, and applied to the identification of historical early synthetic dyestuffs. This is, as far as the authors know, the first study that has successfully applied DESI-MS to historical dye analysis.

DESI is an ambient ionization technique based on electrospray ionization (ESI). A charged spray of solvent is focused onto the sample surface, wetting it so the analyte is desorbed into secondary charged droplets. These charged droplets containing the analyte are directed into the mass spectrometer for analysis using voltage differences (Figure 1).^22,23^ The analysis is rapid, noninvasive, so requires no sample preparation, and minimally destructive, providing significant advantages for applications in dye analysis. However, since it is an ambient technique, it results in more background peaks than non-ambient techniques, which may be a disadvantage if the concentration of the analyte is low. It is also highly dependent on geometrical parameters.^24^ Additionally, it must be noted that DESI-MS is a surface technique, which might be a disadvantage when studying historical objects if much surface contamination is present on the object.

**Figure 1:**
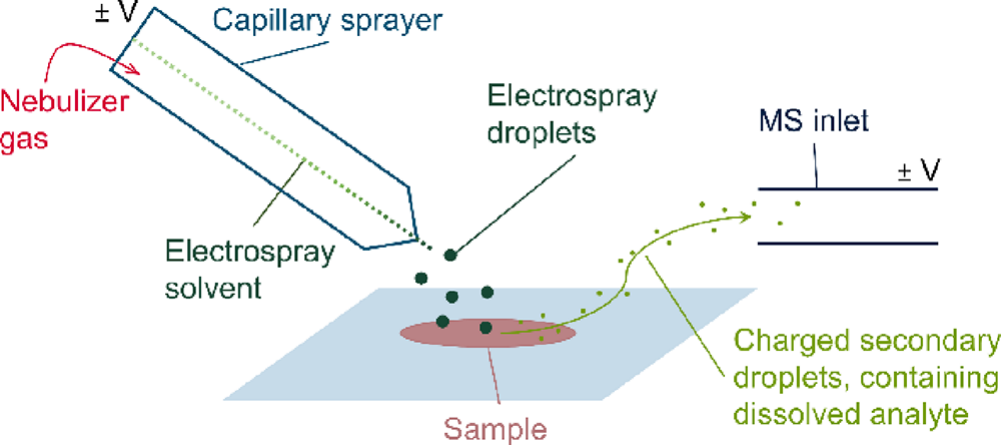
Scheme of the ionization step in DESI-MS analysis.

This study focused on developing DESI-MS for the analysis of early synthetic dyes using both modern and historical references. The historical references were taken from the 1893 edition of Adolf Lehne’s *Tabellarische Übersicht über die künstliche organischen Farbstoffe und ihre Anwendung in Färberei und Zeugdruck* [*Tabular overview of the synthetic organic dyestuffs and their use in dyeing and printing*] (Lehne’s handbook) (Figure 2). Adolf Lehne (1856–1930) was one of the most influential German dye chemists of his time.^25^ Much of his work was focused on the standardization of the emerging and rapidly expanding dyeing industry, both in his own dye laboratories and in an official capacity. He was appointed expert advisor of the textile industry by the Berlin council in 1888 and was part of the Royal Patent Office for textile chemical applications from 1891. During his long chairmanship of “Fachgruppe für Chemie der Farben- und Textilindustrie” [the focus group of dyeing and textile industry chemistry], he developed a German national standardization of dyeing and printing under the “Deutsche Echtheitkommission” [German authenticity commission]. This commission was used as a model for the European international equivalent founded under ISO in 1951. Included in the many important works by Lehne was his handbook, which he printed several editions of and in which he attempted to summarize the most important dyestuffs on the market. The book includes commercial names, dyeing details, and one or more dyed sample(s) of each dyestuff presented (Figure 2). It therefore provides naturally degraded reference samples of early synthetic dyes, making it an important source for understanding original dye recipes and degradation pathways.

**Figure 2:**
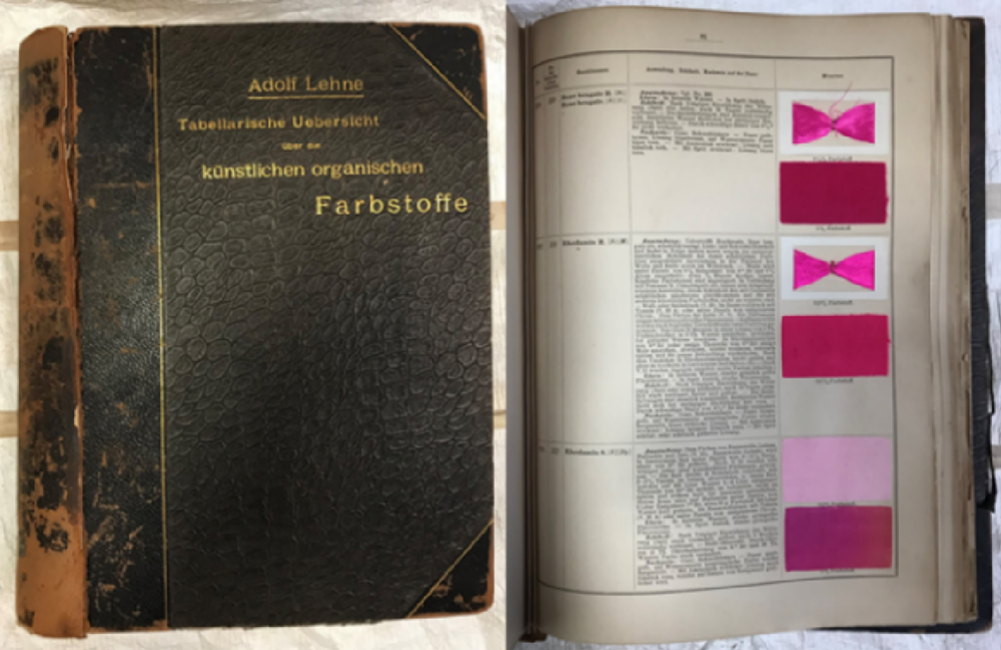
*Tabellarische Übersicht über die künstlichen organischen Farbstoffe und ihre Anwendung in Färberei und Zeugdruck* [*Tabular overview of the synthetic organic dyestuffs and their use in dyeing and printing*], Adolf Lehne, 1893. Page including rhodamine B shown to the right. L. Troalen, private collection.

## Materials and Methods

### Materials

Rhodamine B (CI 45170) (**1**), aniline yellow (CI 11000) (**2**), Congo red (CI 22120) (**3**), orange II sodium salt (CI 15510) (**4**), ponceau S (CI 27195) (**5**), xylidine ponceau (CI 16150) (**6**), auramine O (CI 41000) (**7**), brilliant green (CI 42040) (**8**), malachite green (CI 42000) (**9**), and naphthol yellow S (CI 10316) (**10**) were obtained from Sigma-Aldrich Inc., St. Louis, MO, USA. Methylene blue (CI 52015) (**11**), basic fuchsin (CI 42510) (**12**), methyl violet 2B (CI 42535) (**13**), and Martius yellow (CI 10315) (**14**) were purchased from Fluorochem Ltd., Hadfield, UK. Undyed, degummed, unmordanted silk (2-ply, 66 Tex, thread count 43 cm^–2^) and undyed, washed, unmordanted wool cloth (3-ply, 158 Tex, thread count 36 cm^–2^) from the Monitoring of Damage to Historic Tapestries project (MODHT) (FP5, EC contract number EVK4-CT-2001-00048)^26,27^ and undyed cotton cloth locally purchased (thread count 32 cm^–2^) were used as the reference cloths (microscope images of reference cloths in Figure S1, ESI). The H_2_O, MeOH, and CH_3_CN (ACN) (LC–MS grade) were purchased from Fisher Scientific, Waltham, MA, USA. Dye samples from Lehne’s handbook, 1893 (Figure 2), were also analyzed. Fabric clips (Prym Love clips, 1.0 × 2.6 cm) were bought locally, while water-sensitive paper (Pentair Hypro) was purchased from Agratech NW Ltd., Rossendale, UK.

### Dyeing Procedure

Each reference dyestuff (100 ± 0.005 mg) was dissolved in 7.5 mL of H_2_O, and the dyebaths were heated to 75 °C before 100 ± 0.005 mg (ca. 1 cm^2^) silk cloth was added. The dyebaths were kept at 75 °C for 15 min before the silk samples were removed and rinsed at least twice with cold, deionized water and left to dry completely. The same dyebaths were used to dye the wool samples (150 ± 0.005 mg, ca. 1 cm^2^) following the same procedure, except that the wool samples were pre-wetted in deionized H_2_O for 10 min before dyeing.

### Instrumentation

**Figure 3:**
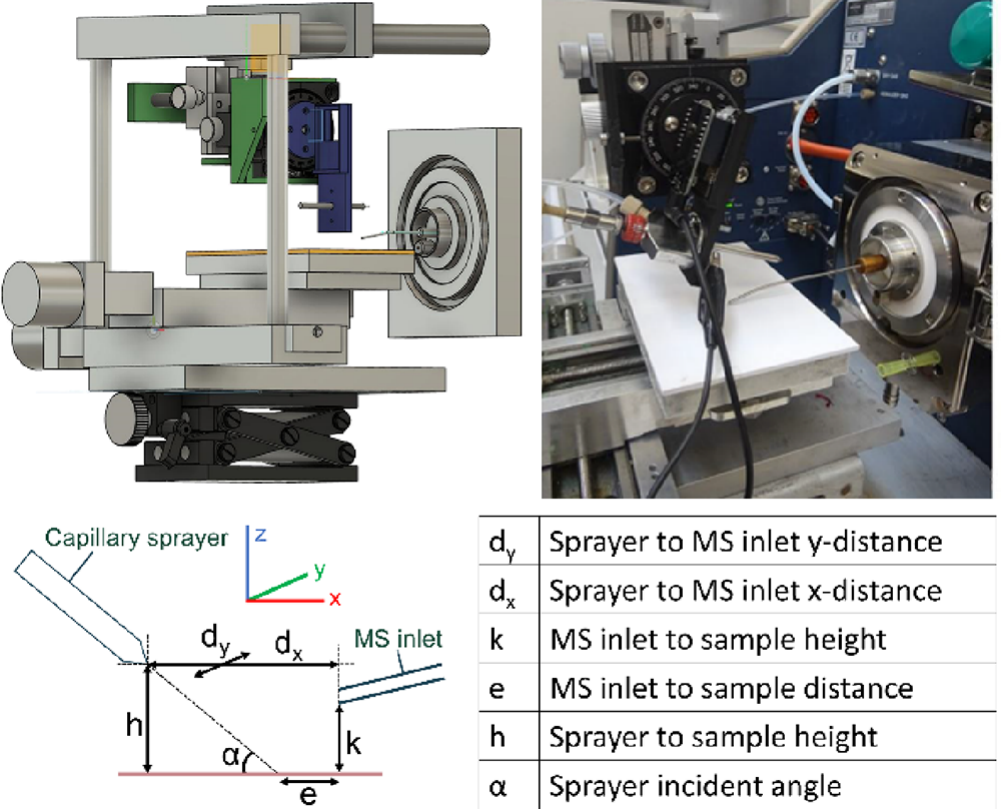
Top left: graphic model of the developed DESI source (Fusion 360, Autodesk, San Francisco, CA, USA). Top right: photograph of the developed DESI source attached to the Bruker 7T SolariX FT-ICR-MS used. Below: geometrical parameters impacting DESI-MS analysis.

A DESI source built in-house (Figure 3) attached to a Bruker 7T SolariX FT-ICR-MS using Compass HyStar 5.1 (Bruker Daltonik GmbH, Billerica, MA, USA) was used for all experiments. The commercial electrospray emitter (part number: 0601815, Bruker Daltonik) used for ESI-MS by the specified mass spectrometer was also utilized by the DESI source. The DESI source was constructed with an acrylic stage mounted on an XY stage controlled via an Arduino-compatible board (EleksMaker EleksMana v5.2) and LaserGRBL (v4.6.0). The sprayer holder was 3-D printed in polylactic acid (Ultimaker 2) and fitted onto a rotation mount (THORLABS RP01 Manual Rotation Stage) attached to three-direction positioners (WPI Inc. Kite Manual Micromanipulator). For spot monitoring, space for a camera (ESP32-CAM) was added above the sprayer tip. The sprayer setup was mounted on stainless-steel rods attached to the stage, and the whole assembly was affixed upon a lab jack for control of the sample–MS inlet distance (*k*; Figure 3). The MS inlet was fitted with a 90 × 0.4 mm (length × i.d.) stainless-steel/brass extension, which was held in place around the MS capillary with a gold spring.

### DESI Analysis

Textile samples (ca. 1 cm^2^) were placed on a glass slide and held in place with fabric clips (Prym Love clips, 1.0 × 2.6 cm). Larger textiles (ca. 10 × 10 cm) were clipped directly onto the plastic stage or, if large enough to not be affected by the nebulizing gas stream (ca. 25 × 25 cm), placed directly on the stage without clipping. The DESI-MS spectra were acquired in the mass range of *m*/*z* 150–1000, except for the analyses of naphthol yellow S (**10**), which used a mass range of *m*/*z* 125–1000 for better visualization of its [M-2Na]^2–^ peak at *m*/*z* 155.98. The following parameters were used for positive mode: capillary voltage, 4.5 kV; end plate offset, −500 V; flow rate, 12.5 μL min^–1^; nebulizing gas, nitrogen; nebulizing gas pressure, 3.9 bar; source temperature, 200 °C. The following parameters were used for negative mode: capillary voltage, 4.2 kV; end plate offset, −800 V; flow rate, 12.5 μL min^–1^; nebulizing gas, nitrogen; nebulizing gas pressure, 3.9 bar; source temperature, 200 °C. The sprayer angle was set at 35°. Two or 10 mass spectra were summed, and the ion accumulation per mass spectrum was 1.5 s. The solvent system used was 3:1 v/v ACN:H_2_O, and the MS inlet was cleaned with LC–MS grade ACN and 3:1 v/v ACN:H_2_O between every analysis. All spectra were processed using Compass DataAnalysis (Bruker Daltonik GmbH, Billerica, MA, USA) and Origin 9.5 (OriginLab, Northampton, MA, USA). Statistical analysis was performed using GraphPad Prism 9.3.1 (GraphPad Software, LLC, San Diego, CA, USA).

## Results and Discussion

### DESI Source Development and Optimization

DESI is highly dependent on multiple parameters, instrumental, geometrical, and chemical in nature, and a small change in one often leads to a necessary change in all others. It is especially dependent on the geometrical parameters of the sprayer setup (Figure 3).^22−24,28,29^ This makes it important to include geometrical control when building the source and the reason manual positioners were incorporated. The sprayer setup block with the positioners and angle mount ended up heavier than expected, so an angle lock and a *z*-direction lock in forms of screws were included in the design (Figure 3).

**Figure 4:**
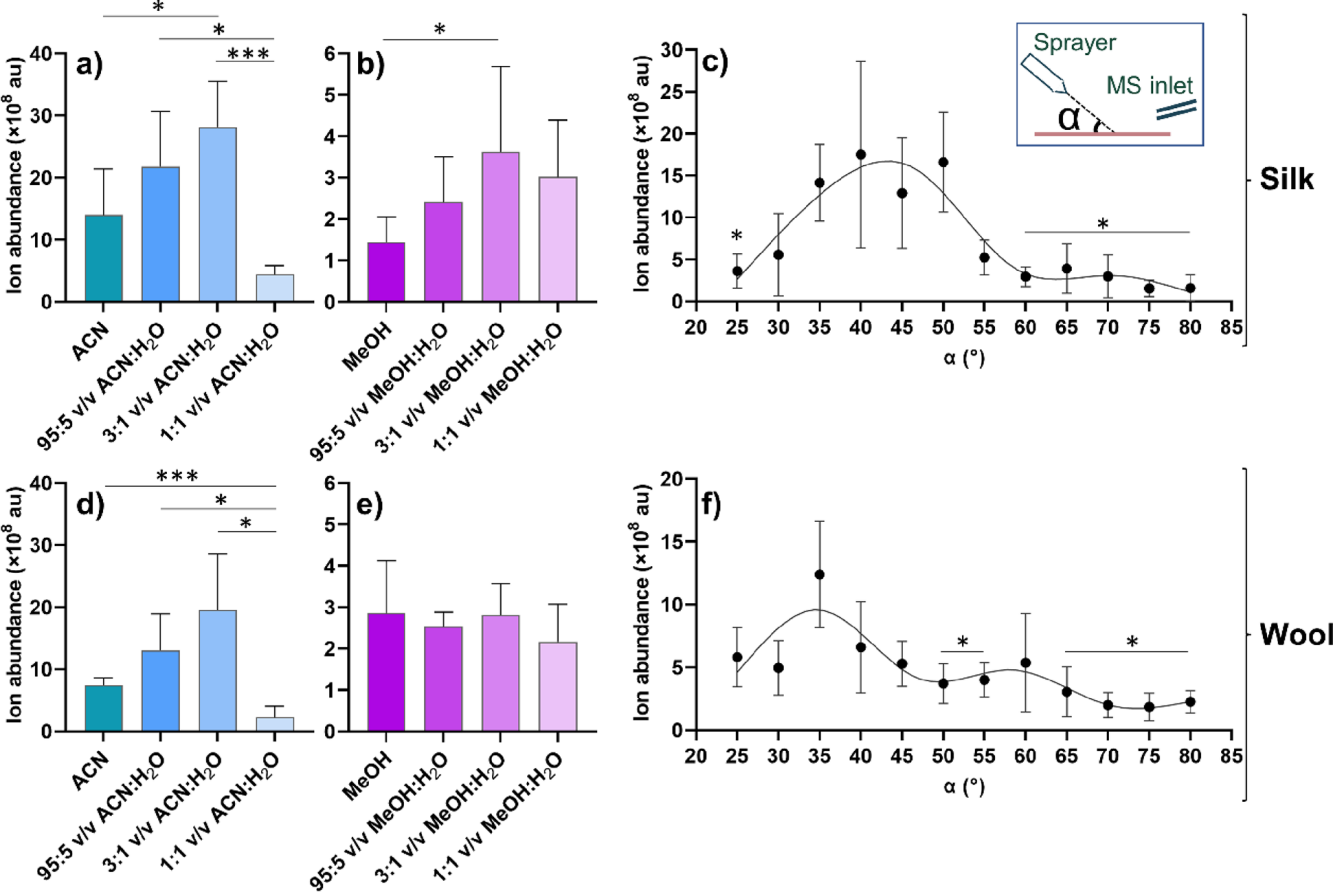
Absolute ion abundance of the [M – Cl]^+^ peak (*m*/*z* 443.23) of rhodamine B (**1**): (a) on silk using four ACN:H_2_O solvent systems (*n* = 6, mean ± S.D.); (b) on silk using four MeOH:H_2_O solvent systems (*n* = 6, mean ± S.D.); (c) on silk using different sprayer incident angles, α (*n* = 7, mean ± S.D.). Inset: A schematic defining the incident sprayer angle, α; (d) on wool using four ACN:H_2_O solvent systems (*n* = 6, mean ± S.D.); (e) on wool using four MeOH:H_2_O solvent systems (*n* = 6, mean ± S.D.); (f) on wool using different sprayer incident angles, α (*n* = 7, mean ± S.D.). * represents *p* values <0.05 and *** *p* values <0.001 calculated using the Brown–Forsythe and Welch ANOVA test. Fitted curves in panels (c) and (f) for data visualization only, constructed as smoothing spline curves using 5 knots (GraphPad Prism 9.3.1).

Rhodamine B (**1**) is commonly used as a standard to optimize DESI setups, due to it being easily ionized, having a characteristic *m*/*z* peak, and being readily available.^24,29^ It is also representative of the early coal-tar dyes that are the focus of this study, by being a basic dye showing high water solubility, strong color, and poor lightfastness.^30^ Additionally, as many historical textiles often contain a mixture of dyes, the development of a general approach applicable to most early synthetic dyes would both take advantage of the rapidness of DESI-MS and facilitate practical use. Therefore, the parameters were optimized using (**1**) but evaluated on a variety of early synthetic dye families. Further studies expanding the use of DESI-MS to other dye classes including natural product, reactive, and metal complex dyes will necessarily need to use other dyes for optimization. Optimization of the DESI source for textile analysis was performed in positive mode, using the [M – Cl]^+^ peak of (**1**) at *m*/*z* 443.23. Initial experiments exploring different nebulizing gas pressures, capillary voltages, geometrical parameters, and solvent systems (Table S1, ESI) suggested that the parameters with the greatest impact on the signal obtained were the solvent system used and the geometrical parameters, particularly the sprayer incident angle (α; Figure 3) and alignment between the sprayer and the MS inlet (*d_y_*; Figure 3). The optimum signal-to-noise ratio was achieved when the MS inlet was as close as possible to the sample, nearly touching it (*k*; Figure 3). The sprayer-to-sample distance (*h*; Figure 3) and sprayer-to-MS inlet in the *x*-direction (*d_x_*; Figure 3) tolerated the most movement of the geometries tested, while even a small misalignment between the sprayer and the MS inlet in the *y*-direction (*d_y_*; Figure 3) meant a complete disappearance of the signal for (**1**). It is uncertain what causes this reliance on *d_y_* alignment, one possibility is the design of the MS inlet extension since its shape was achieved manually, or it could be inherent to the design of the commercial sprayer. The choice of solvents was limited by the conservation context of cultural objects in which only a few solvents are both viewed as acceptable in a museum setting^31^ and MS compatible. Furthermore, additives to the solvent system, such as formic acid and ammonium acetate, have been shown to help with ionization of natural dyestuffs and therefore signal intensity.^32^ However, early synthetic dyes can be sensitive to pH changes and chemical alterations can occur with the addition of acid and base.^33^ Indeed, discoloration of some synthetic dye references was observed (Figure S2, ESI), so additives to the solvent system were not included. Instead, various ratios of LC–MS grade ACN and MeOH with H_2_O were investigated on both silk and wool substrates (Figure 4). Repeat measurements (*n* = 6) were performed at different locations on the same reference sample of silk or wool cloth dyed with (**1**). Each spot location was wetted with the solvent mixture for 1 min before recording since desorption of the analyte into secondary droplets requires the sample surface to be wet.^22,29,34^ The absolute ion abundances used in Figure 4 are the sum of 10 mass spectra each collected after an ion accumulation time of 1.5 s. The sprayer angle (α; Figure 3) was optimized at 35°, *h* at 2 mm, *d_x_* at 4 mm, and *k* at <1 mm for all measurements. The only geometrical parameter changed if necessary was *d_y_* due to the complete disappearance of the signal with any misalignment (*vide supra*). Initial testing of flow rates showed that a higher flow rate was required for textile samples than for biological and forensic samples,^24,28^ as suggested by the literature.^15^ A flow rate of 12.5 μL min^–1^ gave the highest signal-to-noise ratios for the [M – Cl]^+^ peak of (**1**), for the thickness and type of cloth used in this study. The increased flow rate needed in comparison to biological and forensic studies is likely due to the wicking properties of the tightly woven cloths, reducing the formation of the thin liquid layer on the sample surface required for the proposed “droplet pickup” mechanism for analyte transport.^35^ Too low a flow rate resulted in the need for longer analytical times and gave lower ion intensities, while higher flow rates (20–30 μL min^–1^) resulted in the formation of water droplets on the cloth surface, particularly on wool. Figure 4a,b,d,e shows the resulting graphs from the solvent system measurements with Brown–Forsythe and Welch ANOVA test results included to highlight statistical differences between the solvent systems tested. Most notable is the 10-fold higher absolute ion abundance using ACN solvent ratios compared to MeOH ratios for both silk and wool. The overall trend seen in Figure 4a,b,d is an increase in absolute ion abundance with the addition of H_2_O to the organic solvent, culminating at a 3:1 v/v ratio. Only MeOH on wool (Figure 4e) does not follow this trend, suggesting instead that all MeOH:H_2_O ratios tested on wool have a similar impact on the ion abundance obtained. The increase in ion abundance with the addition of H_2_O suggests that some aqueous component is required on both silk and wool; a likely explanation for this phenomenon could be that the rapid evaporation of pure organic solvents does not allow the natural fiber surface to become properly wetted. However, the decrease in ion abundance seen for 1:1 v/v ACN:H_2_O on both silk and wool (Figure 4a,d) in comparison to 3:1 v/v ACN:H_2_O is statistically significant and shows that there is a limit to how much aqueous solvent should be added. MeOH seems to be more tolerant to the addition of H_2_O for both silk and wool (Figure 4b,e). Wool also showed a lower absolute ion abundance in comparison to silk across both ACN and MeOH solvent systems. Both the silk and wool reference cloths were tightly woven with a high thread count with a similar yarn diameter (Figure S1, ESI), so it is likely that the difference in ion intensity is a result of differences in the inherent properties of wool and silk fibers^36^ rather than textile production. Based on these results, 3:1 v/v ACN:H_2_O was determined to be the best solvent system for the substrates and analytes used in this study and was the solvent system used for the following investigations.

**Figure 5:**
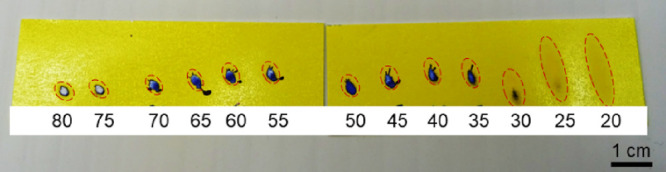
Water-sensitive paper (Pentair Hypro, Agratech NW Ltd., Rossendale, UK) showing the effect of different sprayer incident angles on the solvent (3:1 v/v ACN:H_2_O) spot shape and size. The solvent spot margins are marked by dotted lines.

The next parameter to be optimized was the sprayer angle (α; Figure 3) using 3:1 v/v ACN:H_2_O as the solvent system and the same parameters as the solvent experiments. Shallower angles were shown to give a larger absolute ion intensity (Figure 4c,f) in comparison to steeper angles. The asterisks included show the angles that are statistically different from α = 35° for silk (Figure 4c) and wool (Figure 4f). It must be noted that silk shows a higher ion abundance than wool overall. This is in accordance with the results from the solvent system trials, indicating that silk is better suited for ambient MS analysis than wool. For silk (Figure 4c), there is a clear divide between the lower and higher angles, with the lower angles showing higher ion abundance but also greater standard deviations. This divide is present but not as clear for wool (Figure 4f). That the steeper angles (α = 60° – 80°) are statistically different to α = 35° for both silk and wool suggests that although lower angles for both cloth types give better ion abundances, α = 35° is to be preferred. The large standard deviations seen for both the solvent and angle measurements could perhaps partly be explained by the necessary manual alignment of the *d_y_* parameter as well as the heterogeneity of the dyed rhodamine B references. Despite effort to dye as evenly as possible, it is probable that the dye content varied on a molecular level across the samples analyzed. The increased ion abundance at lower angles, α, is likely due to the increased spot area (Figure 5) and the reported narrower lateral dispersion of secondary droplets using shallower angles.^37^ A larger, more elongated spot is a major disadvantage in imaging and any type of precision work as it increases the risk for cross-contamination. However, the aim for this study was not MS imaging but rather the exploration of DESI identification of historical dyes. The larger spots associated with lower angles could instead be seen as an advantage when analyzing historical objects, as the same volume of solvent spread over a larger area results in a softer impact on the sample, reducing the damage to the object. A larger spot also results in less dependence on the geometrical parameters since *d_y_* becomes marginally more tolerant to misalignment. Such larger, less precise spots can therefore be desired when analyzing historical objects where reducing the impact of the analysis is vital and quantification is not necessarily the objective. Nevertheless, the DESI source constructed includes a movable stage to make future application to imaging possible after optimization of the spot size. However, more damage assessment also needs to be done before covering a larger object surface area.

### Historical Dye Analysis

**Figure 6:**
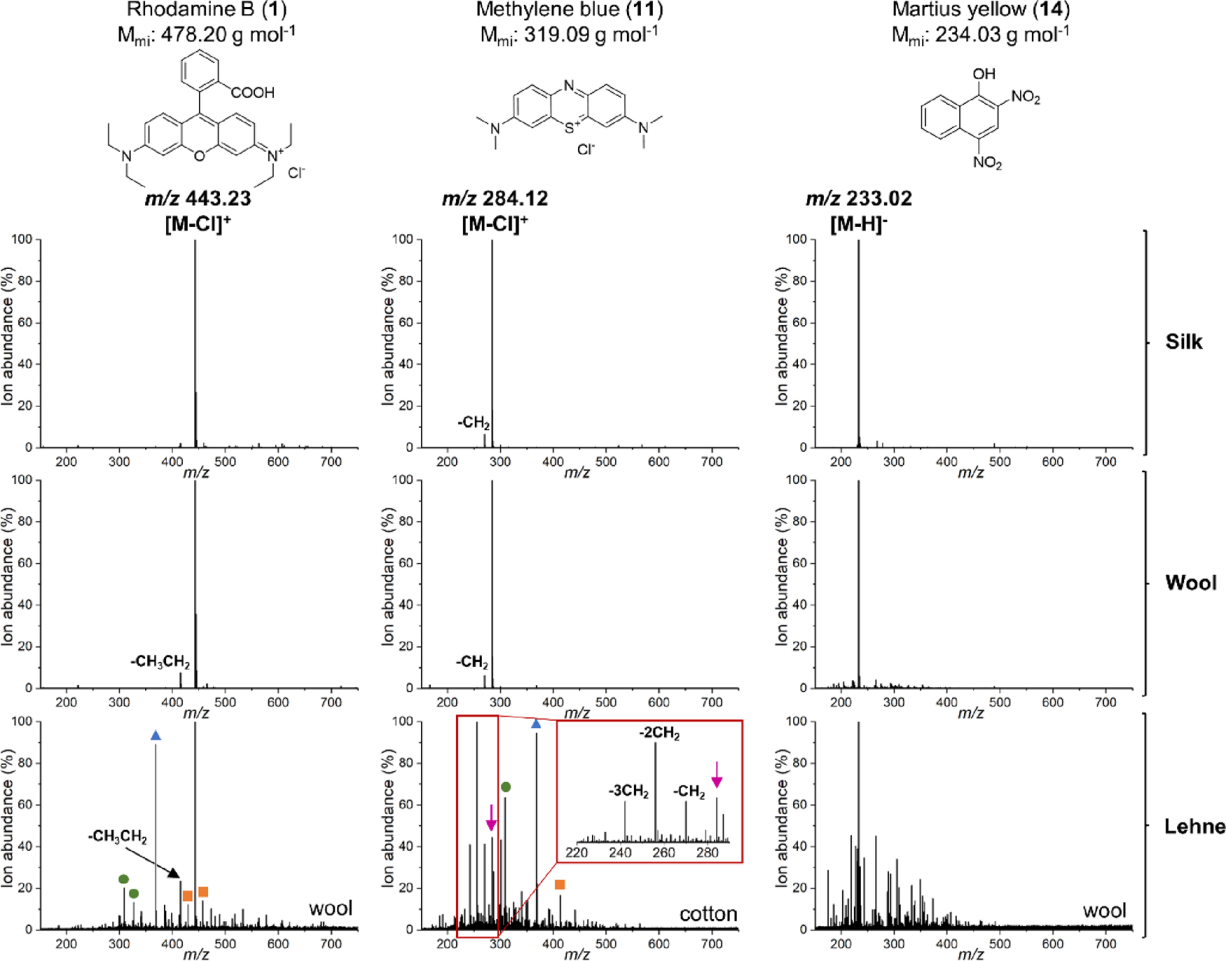
Top: DESI-MS spectra of xanthene rhodamine B (**1**) ([M – Cl]^+^ (*m*/*z* 443.23)), thiazine methylene blue (**11**) ([M – Cl]^+^ (*m*/*z* 284.12)), and nitro dye Martius yellow (**14**) ([M – H]^−^ (*m*/*z* 233.02)) dyed on silk. Centre: DESI-MS spectra of xanthene rhodamine B (**1**) ([M – Cl]^+^ (*m*/*z* 443.23)), thiazine methylene blue (**11**) ([M – Cl]^+^ (*m*/*z* 284.12)), and nitro dye Martius yellow (**14**) ([M – H]^−^ (*m*/*z* 233.02)) dyed on wool. Bottom: DESI-MS spectra of xanthene rhodamine B (**1**) ([M – Cl]^+^ (*m*/*z* 443.23)), thiazine methylene blue (**11**) ([M – Cl]^+^ (*m*/*z* 284.12)), and nitro dye Martius yellow (**14**) ([M – H]^−^ (*m*/*z* 233.02)) from historical samples in Lehne’s handbook (1893). Marked with a blue triangle is *m*/*z* 368.43 (BTAC-228), and green circle is *m*/*z* 309.21 (PPG) and *m*/*z* 327.18 (PEG). An orange square marks phthalates at *m*/*z* 301.07, 413.26, 429.24, and 457.27. The [M – Cl]^+^ (*m*/*z* 284.12) of (**11**) in the Lehne sample is indicated with an arrow, and the spectrum includes a zoomed-in inset to show the degradation products and synthetic byproducts of (**11**). Sum of two mass spectra shown.

**Figure 7:**
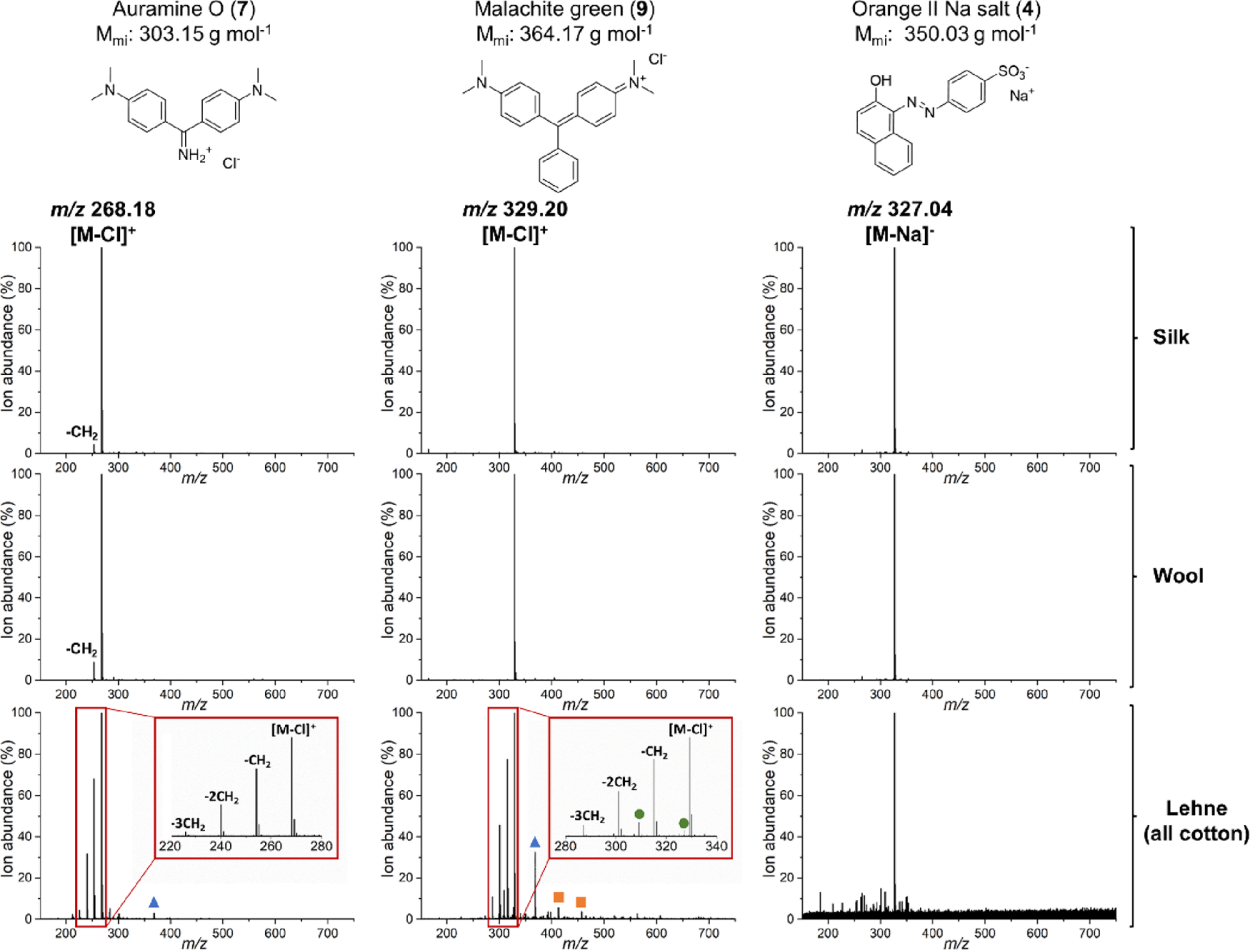
Top: DESI-MS spectra of diphenylmethane auramine O (**7**) ([M – Cl]^+^ (*m*/*z* 268.18)), triphenylmethane malachite green (**9**) ([M – Cl]^+^ (*m*/*z* 329.20)), and azo dye orange II sodium salt (**4**) ([M – Na]^−^ (*m*/*z* 327.04)) dyed on silk. Centre: DESI-MS spectra of diphenylmethane auramine O (**7**) ([M – Cl]^+^ (*m*/*z* 268.18)), triphenylmethane malachite green (**9**) ([M – Cl]^+^ (*m*/*z* 329.20)), and azo dye orange II sodium salt (**4**) ([M – Na]^−^ (*m*/*z* 327.04)) dyed on wool. Bottom: DESI-MS spectra of diphenylmethane auramine O (**7**) ([M – Cl]^+^ (*m*/*z* 268.18)), triphenylmethane malachite green (**9**) ([M – Cl]^+^ (*m*/*z* 329.20)), and azo dye orange II sodium salt (**4**) ([M – Na]^−^ (*m*/*z* 327.04)) from historical samples in Lehne’s handbook (1893). Marked with a blue triangle is *m*/*z* 368.43 (BTAC-228), and green circle is *m*/*z* 309.21 (PPG) and *m*/*z* 327.18 (PEG). An orange square marks phthalates at *m*/*z* 301.07, 413.26, 429.24, and 457.27. The Lehne sample spectra for (**7**) and (**9**) include a zoomed-in inset to show the degradation products and synthetic byproducts of the dyestuffs. Sum of two mass spectra shown.

**Figure 8:**
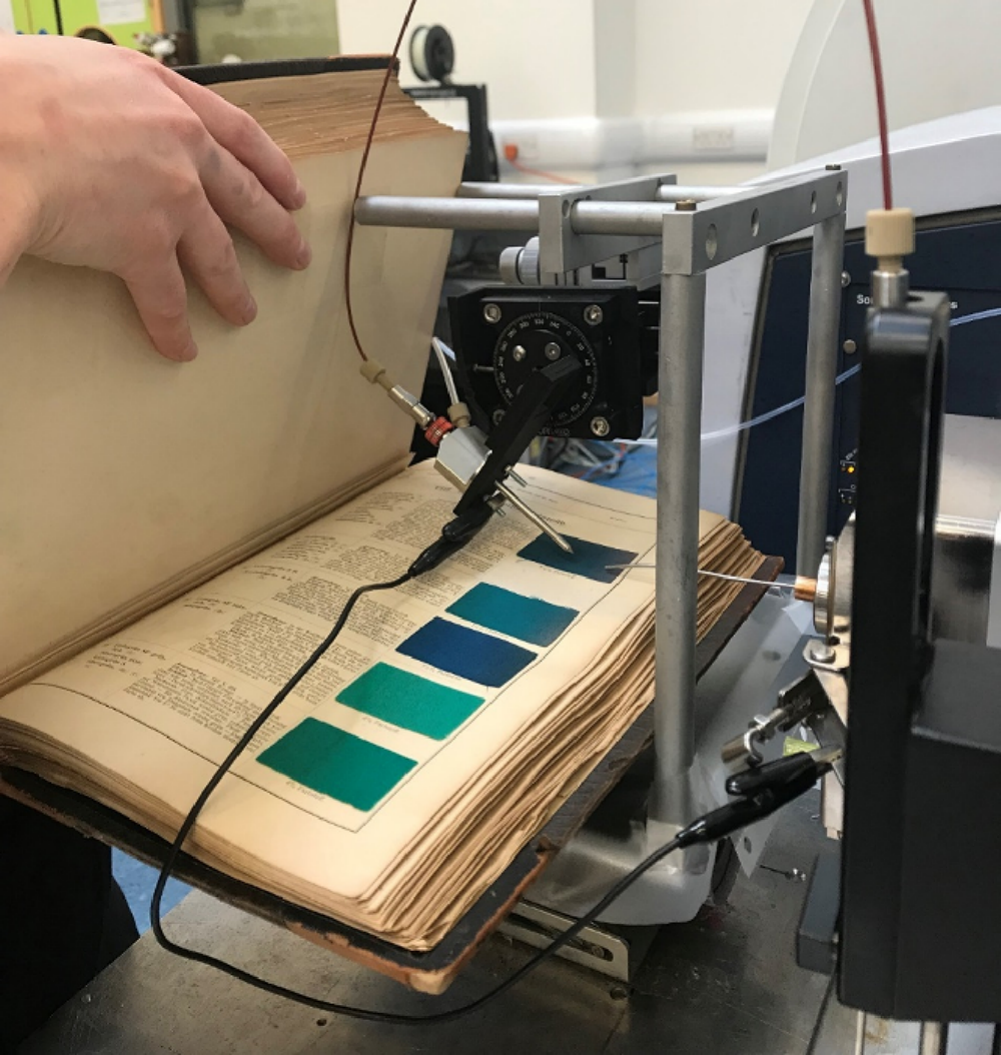
*In situ* analysis of historical samples from Lehne’s handbook (1893). Malachite green (**9**) is being analyzed in the photograph.

After optimization, modern references as well as historical samples from Lehne’s handbook (1893) were analyzed using 35° sprayer angle, α, and 3:1 v/v ACN:H_2_O as the solvent system. The references used covered six important early synthetic dye families, xanthene, thiazine, nitro, diphenylmethane, triphenylmethane, and azo, which are all derivatives of coal-tar compounds and among the first dye compounds invented following Perkin’s serendipitous discovery of mauveine in 1856.^1^ Before dyeing unmordanted silk and wool, conventional ESI-MS using the same FT-ICR-MS instrument was performed on the dyebaths as a comparison to the DESI-MS result and to give a guide for which *m*/*z* peaks to expect for each sample. Of the 14 compounds, 8 were analyzed in positive mode, including the xanthene, thiazine, diphenylmethane, triphenylmethanes, and one azo dye ((**1**), (**2**), (**7**), (**8**), (**9**), (**11**), (**12**), and (**13**)), and the remaining 6 were analyzed in negative mode, including the nitro dyes and all other azo dyes ((**3**), (**4**), (**5**), (**6**), (**10**), and (**14**)). The spectra for all dyestuffs are shown in the Supporting Information together with the MS spectra of undyed silk, wool, and cotton cloths. Figures 6 and 7 show the DESI-MS spectra of an example from each dye family dyed on silk and wool and from Lehne’s handbook (1893). Due to the rapid and direct nature of DESI-MS analysis, the book could be manually held open during the analysis (Figure 8). The dye samples included in Lehne’s handbook were dyed in Lehne’s own laboratory following dyeing procedures detailed adjacent to the cloth (Figures 1 and 8). Their inclusion makes the book an invaluable resource for the study of early synthetic dyes as they give the chemical composition of late 19th century dye recipes and an insight to naturally occurring degradation pathways. All compounds in Figures 6 and 7 except Martius yellow (**14**) are commercially sold as salts, meaning that the characteristic peak seen was [M – Cl]^+^ for the cationic dyes and [M – Na]^−^ for the anionic dyes. The presence of charged species makes the ionization step more efficient, accounting for the clean spectra seen despite DESI-MS being an ambient technique. However, the applicability of DESI-MS to dyestuffs readily ionized at neutral pH is shown by the equally clean spectra of (**14**) in both reference and historical samples, with *m*/*z* 233.02 corresponding to [M – H]^−^ (Figure 6), and the historical sample of picric acid, with *m*/*z* 227.99 corresponding to [M – H]^−^ (Table S3, ESI). Consistently, DESI-MS analysis in positive ion mode gave higher ion abundances than analysis in negative ion mode, and the analyses of the modern dyed reference samples on both silk and wool show little background interference despite being performed under ambient conditions.

For the Lehne samples, the lower concentrations of the dyestuffs resulted in more intense background peaks. Particularly in positive mode, the *m*/*z* peak at 368.43 (blue triangle, Figures 6 and 7), identified as BTAC-228, a known additive in hygiene products, PPG at *m*/*z* 309.21 and PEG at *m*/*z* 327.18 (green circles, Figures 6 and 7), as well as phthalates at *m*/*z* 301.07, 413.26, 429.24, and 457.27 (orange squares, Figures 6 and 7)^38,39^ were difficult to remove. The presence of consistent contaminants highlights the effect of the laboratory environment, which needs to be taken into consideration when working with low concentrations. It is therefore necessary to confidently identify common background peaks since it is difficult to control an open laboratory environment. Regardless of the increased background, the *m*/*z* of the dyestuffs can still be clearly identified in the historical Lehne samples, which demonstrates the applicability of DESI-MS for dye analysis despite it being an ambient technique. The DESI-MS analyses were sensitive enough that degradation products of the dyestuffs studied could be seen when comparing the Lehne samples with the modern reference samples; in particular, the *N*-demethylation of di- and triphenylmethane dyes, which result from known degradation pathways for these dyes.^40−42^ This pattern can be seen for both auramine O (**7**) and malachite green (**9**) (Figure 6a,b). A similar pattern of −CH_2_ and −CH_2_CH_2_ groups can also be seen for xanthene rhodamine B (**1**) and thiazine methylene blue (**11**) (Figure 5a,b) as well as for brilliant green (Table S3, ESI), demonstrating that DESI-MS is sufficiently sensitive for historical dye analysis. A more detailed analysis of the DESI-MS spectra collected for auramine O (**7**) as enabled by the use of the FT-ICR analyzer (Figure S3, ESI) reveals the presence of both isotopic and contaminant *m*/*z* values for the parent ion and each of the corresponding demethylation products. These arise from the parallel decomposition pathways for auramine O and its synthetic intermediate and hydrolysis product, Michler’s ketone.^43^ The observation of these species with close *m*/*z* values highlights the value of high-resolution MS in the absence of chromatographic separation and suggests that in future, synthetic route investigations could be conducted using the developed workflow. It showcases again the potential applications of DESI-MS to the field of dye analysis.

## Conclusions

A DESI-MS source has been built in-house to allow the noninvasive analysis of historical dyes. The setup used the ESI sprayer attached to a Bruker 7T SolariX FT-ICR-MS instrument and included *x*-, *y*-, and *z*-positioners and an angle mount for manual control of the geometrical parameters. Optimization of the geometrical and chemical parameters was carried out in positive mode on silk and wool samples dyed with rhodamine B to show that a sprayer incident angle, α, of 35° and a solvent system of 3:1 v/v ACN:H_2_O gave the highest absolute ion abundance for analysis on both silk and wool. This setup was used with success on 14 early synthetic dyes covering six important chemical families, showing the applicability of DESI-MS for dye analysis. Dye references from 1893 were also investigated with success using DESI-MS, in which natural degradation could be clearly seen. Further damage assessment and studies on more complex systems are currently underway, but the advantage of rapid, *in situ* MS analysis in the field of dye analysis cannot be stressed enough. This development of DESI-MS for historical dye analysis will hopefully give access to valuable information on *hitherto* inaccessible objects.

## Supporting Information Available

Table of parameter ranges tested during optimization; DESI-MS spectra of background (glass slide), silk, wool, and cotton cloths; DESI-MS spectra of compounds **1**–**14** on silk and wool as well as DESI-MS spectra of **1**, **4**, **7–9**, **11–14** and picric acid from Lehne’s handbook (1893); images of damage in the presence and absence of solvent additives; and DESI-MS spectra for auramine O showing isotopic and synthetic contaminant peaks (PDF)

## Notes

Primary data files can be found at https://doi.org/10.7488/ds/3816.
