# Supplementary material for: Development and Application of Desorption Electrospray Ionization Mass Spectrometry for Historical Dye Analysis
Source: Anal Chem. 2023 Mar 1;95(11):4846–54. doi: 10.1021/acs.analchem.2c03281 (PMC10034744; doi:10.1021/acs.analchem.2c03281)
Supplement: Supplementary file 1 — ac2c03281_si_001.pdf [file ac2c03281_si_001.pdf]

Supporting Information

Development and application of desorption electrospray ionisation mass spectrometry (DESI-MS) for historical dye analysis

Edith Sandström,[a,b] Chiara Vettorazzo,[a] C. Logan Mackay,[a] Lore G. Troalen[b] and Alison N. Hulme [a,*]

[a.] EaStCHEM School of Chemistry, University of Edinburgh, David Brewster Road, Edinburgh, EH9 3FJ, UK.
[b.] National Museums Scotland, Department of Collections Services, National Museums Collection Centre, 242 West Granton Road, Edinburgh, EH5 1JA, UK.

Page	Contents
S2	Figure S1: Digital microscopy images of silk, wool and cotton cloth references.
S2	Table S1: Ranges and parameters tested during initial experiments.
S3	Table S2: DESI-MS spectra of background cloth in both negative and positive mode.
S4	Table S3: DESI-MS spectra of 14 early synthetic dyes from 6 dye families.
S10	Figure S2: Comparison of discoloration of the azo dye aniline yellow using solvent system containing additives or not.
S11	Figure S3: Auramine O from Lehne's handbook (1893) highlighting the M+1 peaks and likely Michler's ketone peaks for demethylation products.

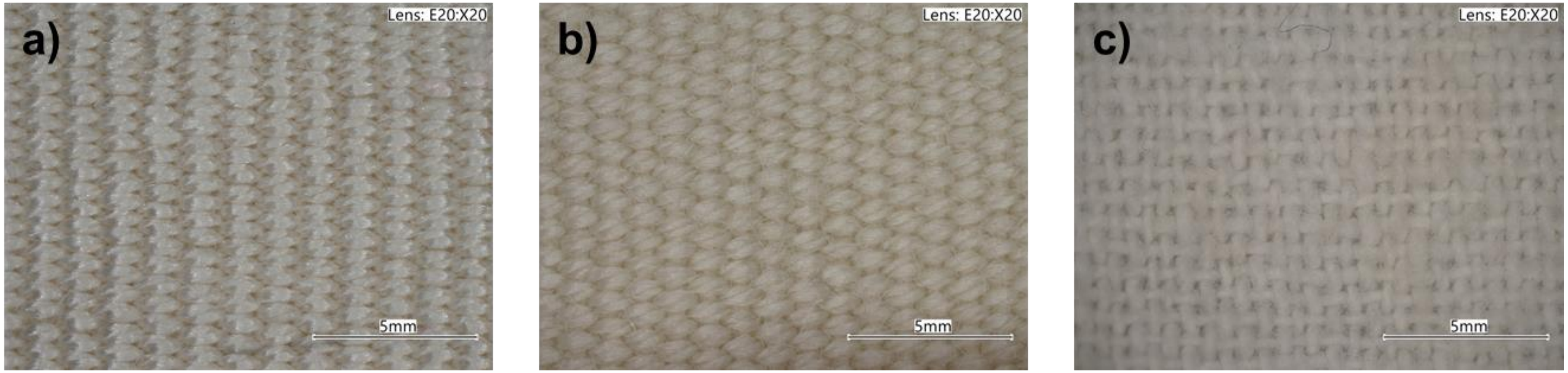

Figure S1. a) Digital microscope image of silk cloth reference. **b)** Digital microscope image of wool cloth reference. **c)** Digital microscope image of cotton cloth reference.

Table S1. MS parameters tested for their effect on the absolute ion abundance of Rhodamine B $[M\text{-}Cl]^+$ peak (*m/z* 443.23), the range tested and the optimised ranges.

MS parameter	Range tested	Range for greatest absolute ion abundance (*m/z* 433.23) (n = 3)
Sweep excitation energy	12 – 15 %	15 %
Skimmer voltage	5 – 30 V	15 – 25 V
Source temperature	200 – 300 °C	250 – 300 °C
Sprayer capillary voltage	1 – 5 kV	4 – 4.5 kV
Dry gas flow	1.0 – 5.0 bar	3.5 – 4.0 bar
Accumulation	0.2 – 3.0 s	1.5 – 2.5 s
Time-of-flight (ToF)	0.2 – 0.8 ms	0.5 – 0.7 ms
Flow rate	3.3 – 33.3 µL min^{-1}	11.7 – 13.3 µL min^{-1}

Table S2. DESI-MS spectra of background cloth in both negative and positive mode. Sum of 2 mass spectra shown and the *m/z* values of the major peaks are added.

Marked with a blue triangle is *m/z* 368.43 (BTAC-228), green circle is *m/z* 309.21 (PPG) and *m/z* 327.18 (PEG). An orange square marks phthalates at *m/z* 301.07, 413.26, 429.24, 457.27.

<table>
<tr><td>Background, glass slide, positive mode</td><td>Background, glass slide, negative mode</td></tr>
<tr><td>
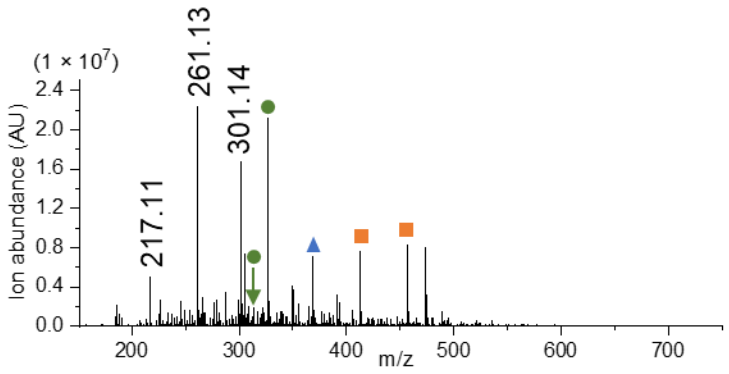

</td><td>
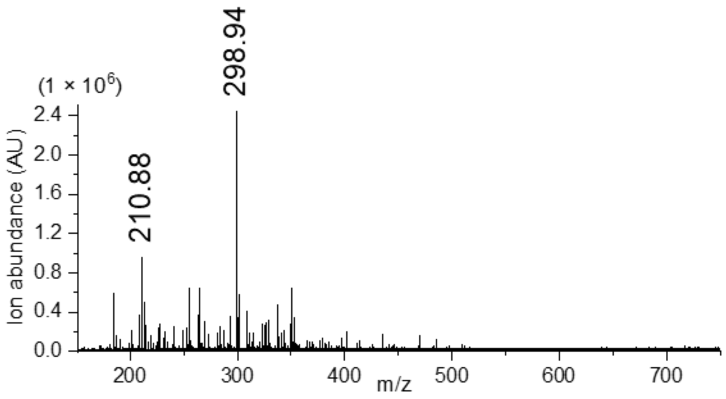

</td></tr>
<tr><td>Undyed silk, positive mode</td><td>Undyed silk, negative mode</td></tr>
<tr><td>
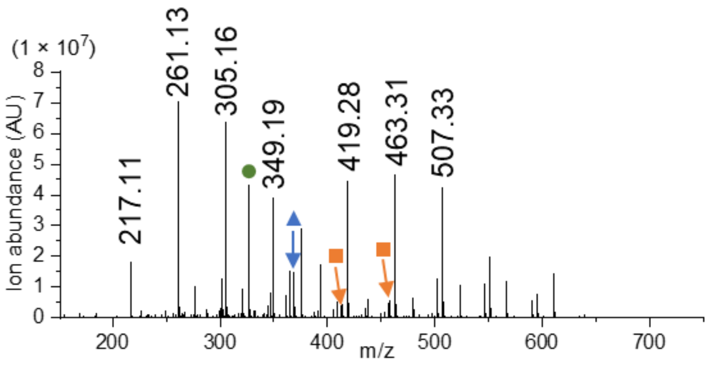

</td><td>
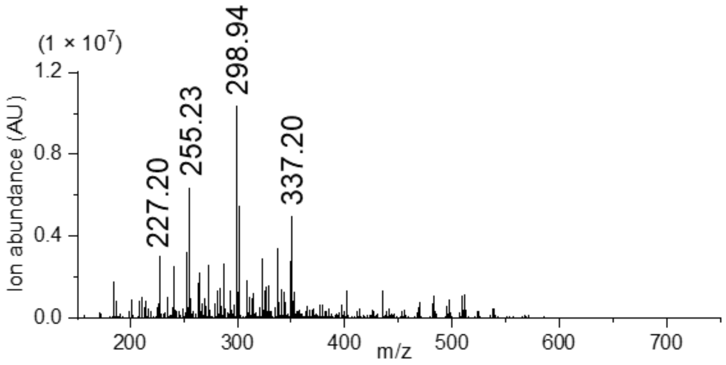

</td></tr>
</table>

Table S2 continued.

<table>
<tr><td>Undyed wool, positive mode</td><td>Undyed wool, negative mode</td></tr>
<tr><td>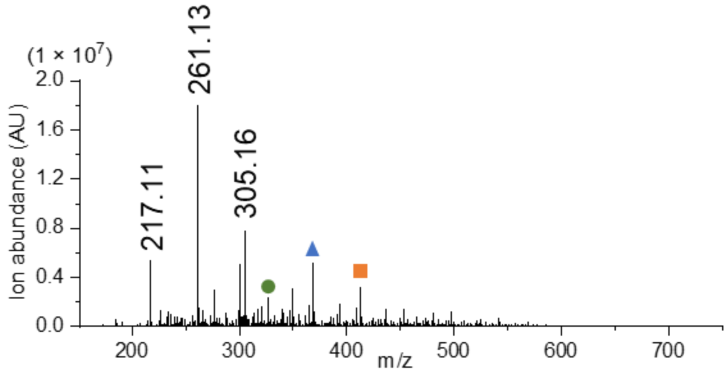
</td><td>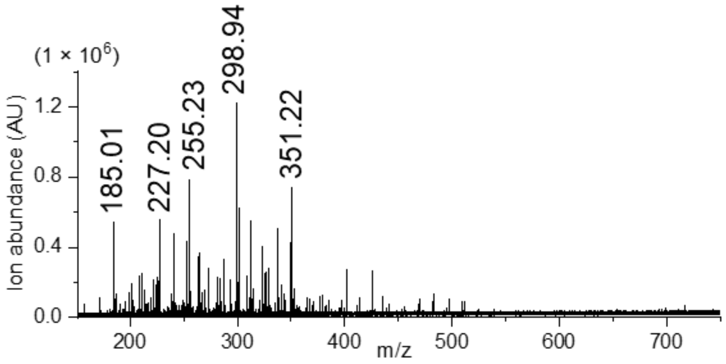
</td></tr>
<tr><td>Undyed cotton, positive mode</td><td>Undyed cotton, negative mode</td></tr>
<tr><td>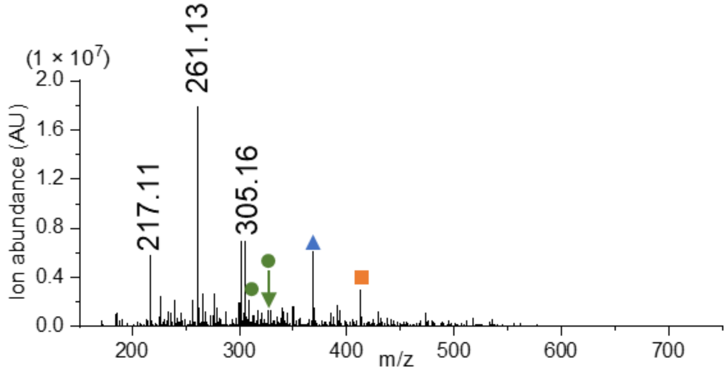
</td><td>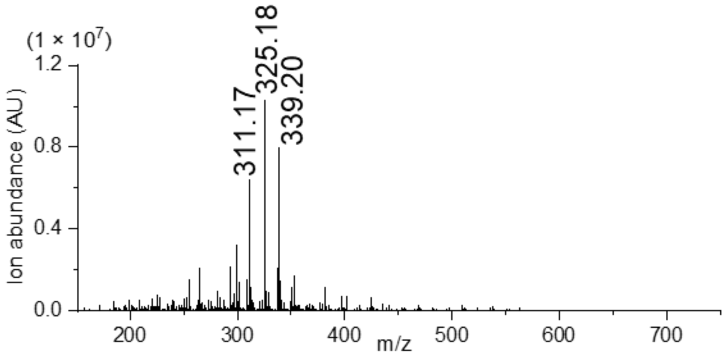
</td></tr>
</table>

Table S3. DESI-MS spectra of 14 early synthetic dyes from 6 dye families. Sum of two mass spectra shown; where needed the molecular ion is marked with a pink arrow.

Marked with a blue triangle is *m/z* 368.43 (BTAC-228), green circle is *m/z* 309.21 (PPG) and *m/z* 327.18 (PEG). An orange square marks phthalates at *m/z* 301.07, 413.26, 429.24, 457.27.

Compound (CI number) Family	Structure	M_{mi} (g mol^{-1})	Characteristic ion	DESI-MS spectrum Silk	DESI-MS spectrum Wool	DESI-MS spectrum Lehne sample
Aniline Yellow (CI 11000) Azo	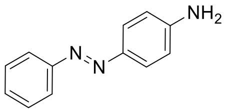	197.10	*m/z* 198.10 [M+H]$^+$	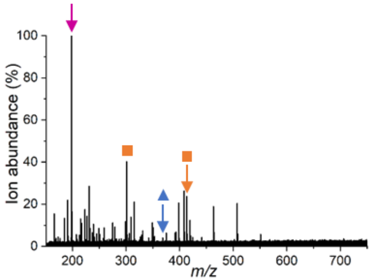	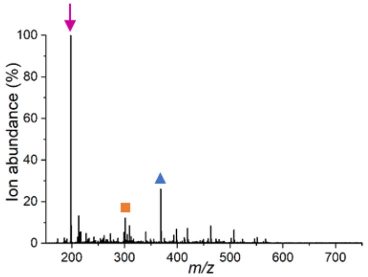	
Congo Red (CI 22120) Azo	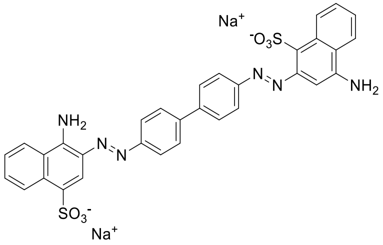	696.08	*m/z* 325.18 [M-Na]$^{2-}$	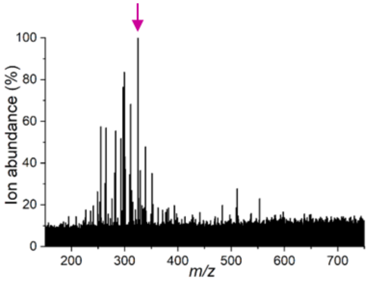	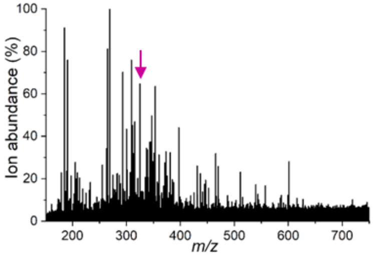	
Orange II Na salt (CI 15510) Azo	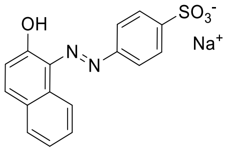	350.03	*m/z* 327.04 [M-Na]$^-$	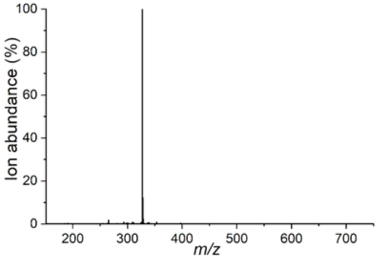	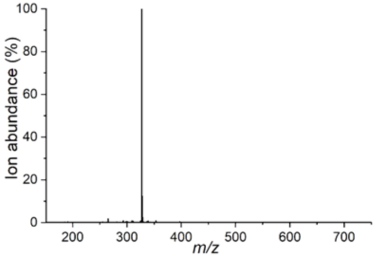	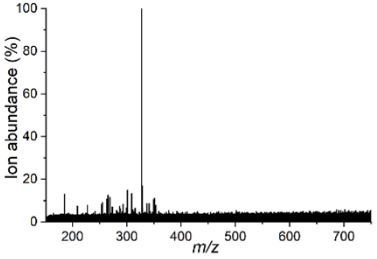

Table S3 continued.

Compound (CI number) Family	Structure	M_{mi} (g mol^{-1})	Characteristic ion	DESI-MS spectrum Silk	DESI-MS spectrum Wool	DESI-MS spectrum Lehne sample
Ponceau S (CI 27195) Azo	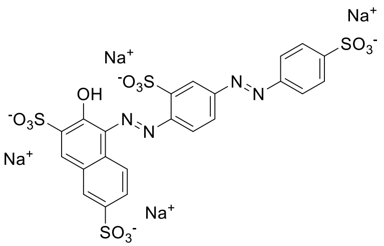	759.89	(i) *m/z* 356.96 $[M-2Na]^{2-}$ (ii) *m/z* 230.31 $[M-3Na]^{3-}$ (iii) *m/z* 166.98 $[M-4Na]^{4-}$	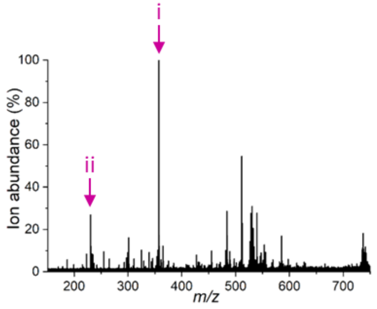	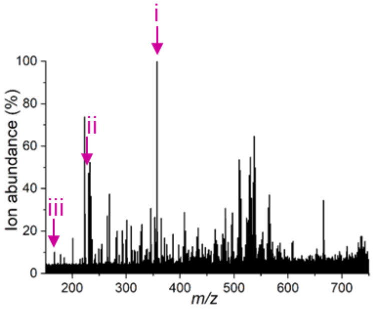	
Xylidine Ponceau (Ponceau 2R) (CI 16150) Azo	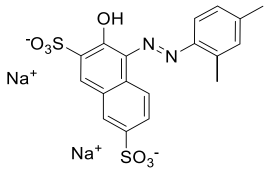	480.00	(i) *m/z* 457.02 $[M-Na]^{-}$ (ii) *m/z* 217.01 $[M-2Na]^{2-}$	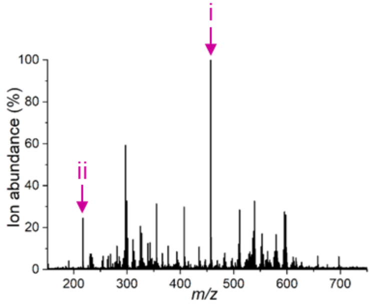	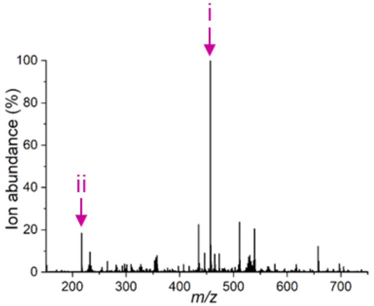	
Auramine O (CI 41000) Diphenyl-methane	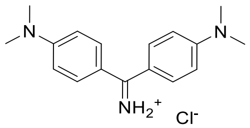	303.15	*m/z* 268.18 $[M-Cl]^{+}$	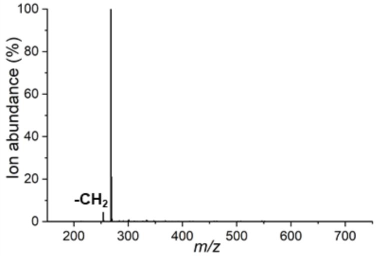	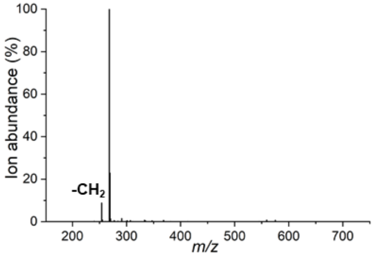	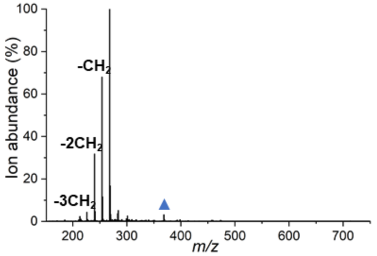

Table S3 continued.

Compound (CI number) Family	Structure	M_{mi} (g mol^{-1})	Characteristic ion	DESI-MS spectrum Silk	DESI-MS spectrum Wool	DESI-MS spectrum Lehne sample
Brilliant Green (CI 42040) Triphenyl-methane	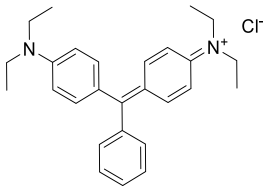	420.23	*m/z* 385.27 $[M-Cl]^+$	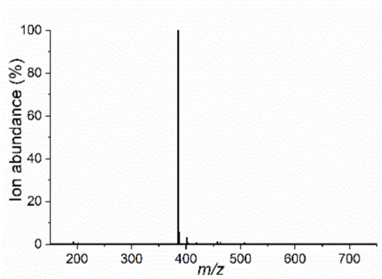	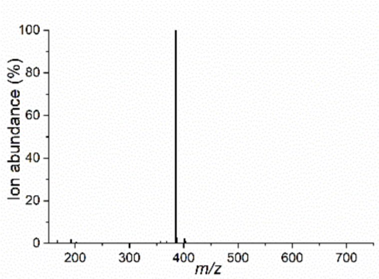	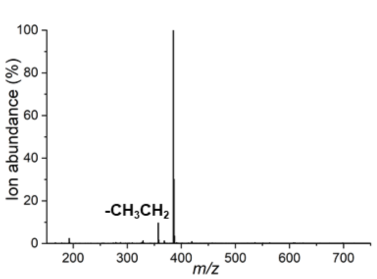
Malachite Green (CI 42000) Triphenyl-methane	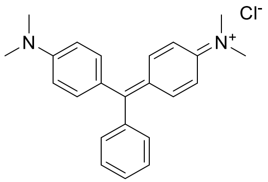	364.17	*m/z* 329.20 $[M-Cl]^+$	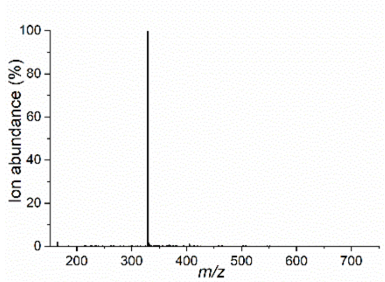	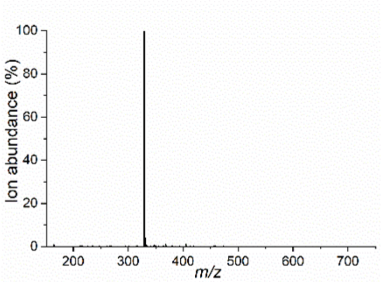	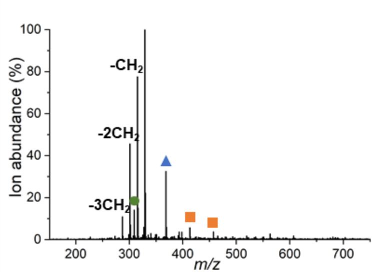
Methyl Violet (CI 42535) Triphenyl-methane	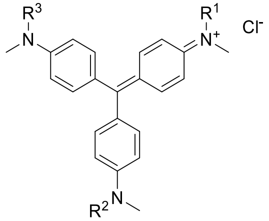 (i) $R^1 = H$, $R^2 = H$, $R^3 = H$<br>(ii) $R^1 = H$, $R^2 = CH_3$, $R^3 = H$<br>(iii) $R^1 = H$, $R^2 = CH_3$, $R^3 = CH_3$<br>(iv) $R^1 = CH_3$, $R^2 = CH_3$, $R^3 = CH_3$	(i) 365.17<br>(ii) 379.18<br>(iii) 393.20<br>(iv) 407.21	(i) *m/z* 330.20 $[M-Cl]^+$<br>(ii) *m/z* 344.22 $[M-Cl]^+$<br>(iii) *m/z* 358.23 $[M-Cl]^+$<br>(iv) *m/z* 372.25 $[M-Cl]^+$	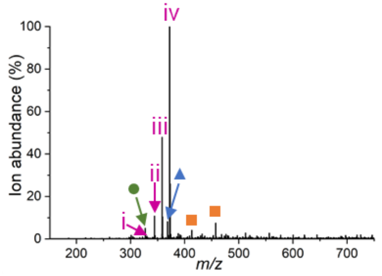	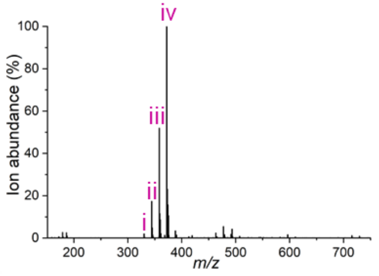	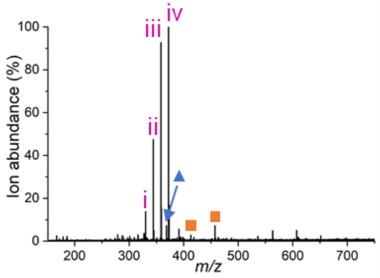

Table S3 continued.

Compound (CI number) Family	Structure	M_{mi} (g mol^{-1})	Characteristic ion	DESI-MS spectrum Silk	DESI-MS spectrum5 Wool	DESI-MS spectrum Lehne sample
Basic fuchsin (CI 42510) Triphenyl-methane	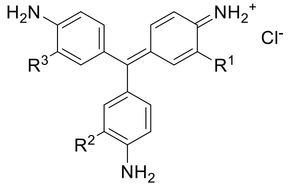 (i) $R^1 = H$, $R^2 = H$, $R^3 = H$<br>(ii) $R^1 = H$, $R^2 = CH_3$, $R^3 = H$<br>(iii) $R^1 = H$, $R^2 = CH_3$, $R^3 = CH_3$<br>(iv) $R^1 = CH_3$, $R^2 = CH_3$, $R^3 = CH_3$	(i) 323.12<br>(ii) 337.14<br>(iii) 351.15<br>(iv) 365.17	(i) *m/z* 288.15 $[M-Cl]^+$<br>(ii) *m/z* 302.17 $[M-Cl]^+$<br>(iii) *m/z* 316.18 $[M-Cl]^+$<br>(iv) *m/z* 330.20 $[M-Cl]^+$	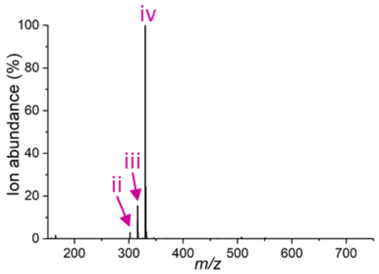	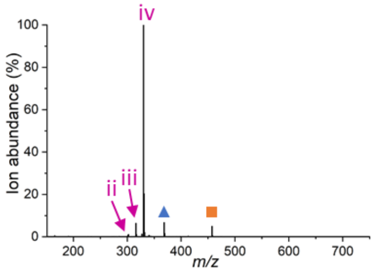	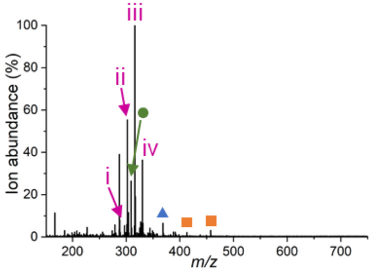
Martius yellow (CI 10315) Nitro	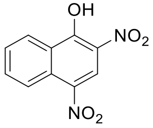	234.03	*m/z* 233.02 $[M-H]^-$	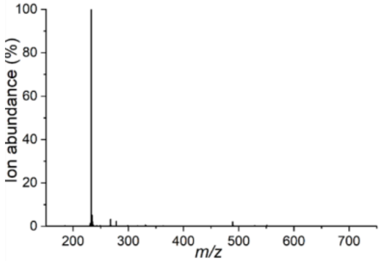	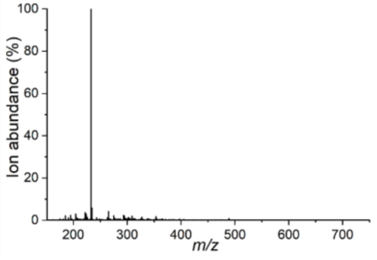	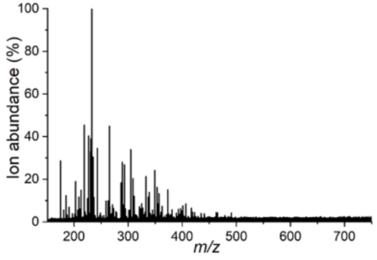
Naphthol Yellow S (CI 10316) Nitro	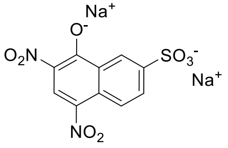	357.95	*m/z* 155.98 $[M-2Na]^{2-}$	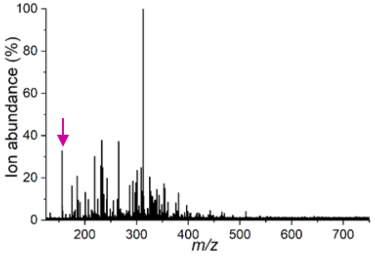	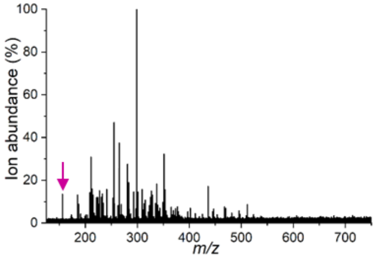	

Table S3 continued.

Compound (CI number) Family	Structure	M_{mi} (g mol^{-1})	Characteristic ion	DESI-MS spectrum Silk	DESI-MS spectrum Wool	DESI-MS spectrum Lehne sample
Picric acid (CI 10305) Nitro	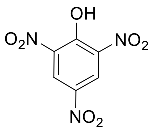	229.00	*m/z* 227.99 $[M-H]^-$			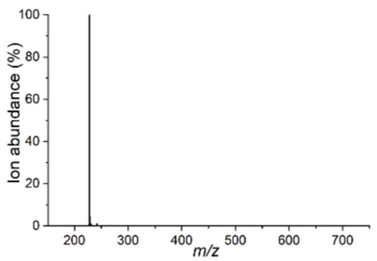
Rhodamine B (CI 45170) Xanthene	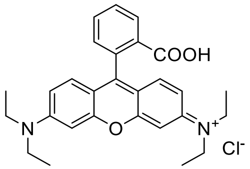	478.20	*m/z* 443.23 $[M-Cl]^+$	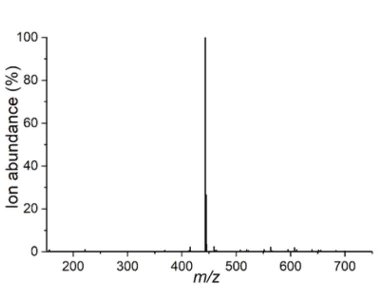	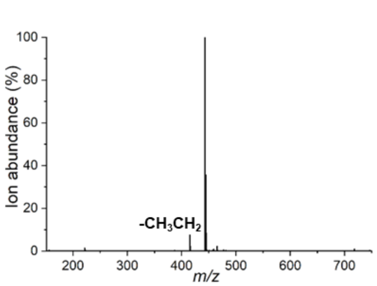	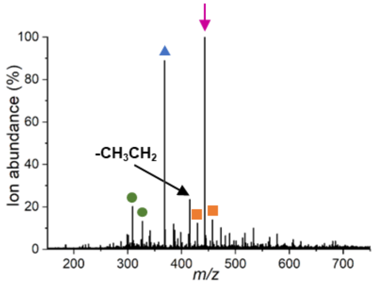
Methylene Blue (CI 52015) Thiazine	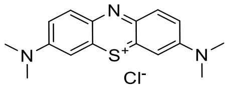	319.09	*m/z* 284.12 $[M-Cl]^+$	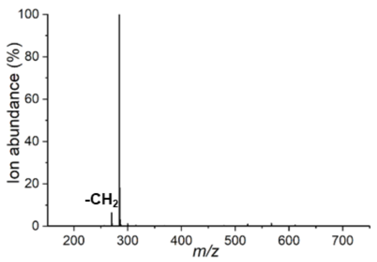	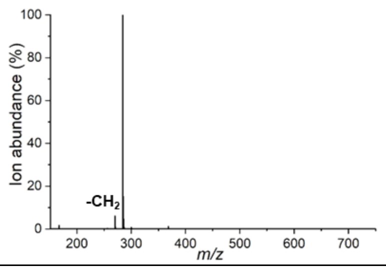	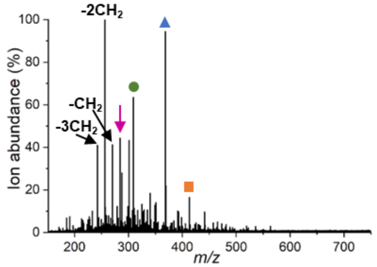

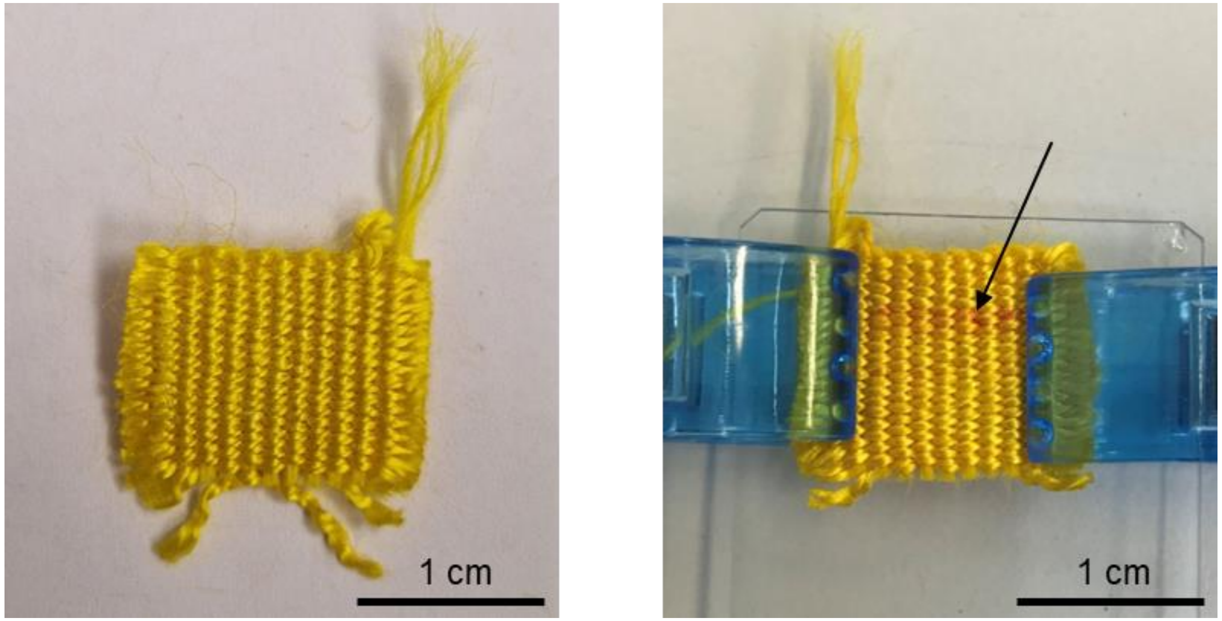

Figure S2. Left: Silk cloth dyed with azo dye aniline yellow after repeated DESI-MS analysis of solvent systems containing no additives. Right: Silk cloth dyed with azo dye aniline yellow after DESI-MS analysis using a solvent system containing an additive (1:1 v/v ACN:H2O + 0.1 % ammonium acetate (1 M)). The discolouration is highlighted with a black arrow.

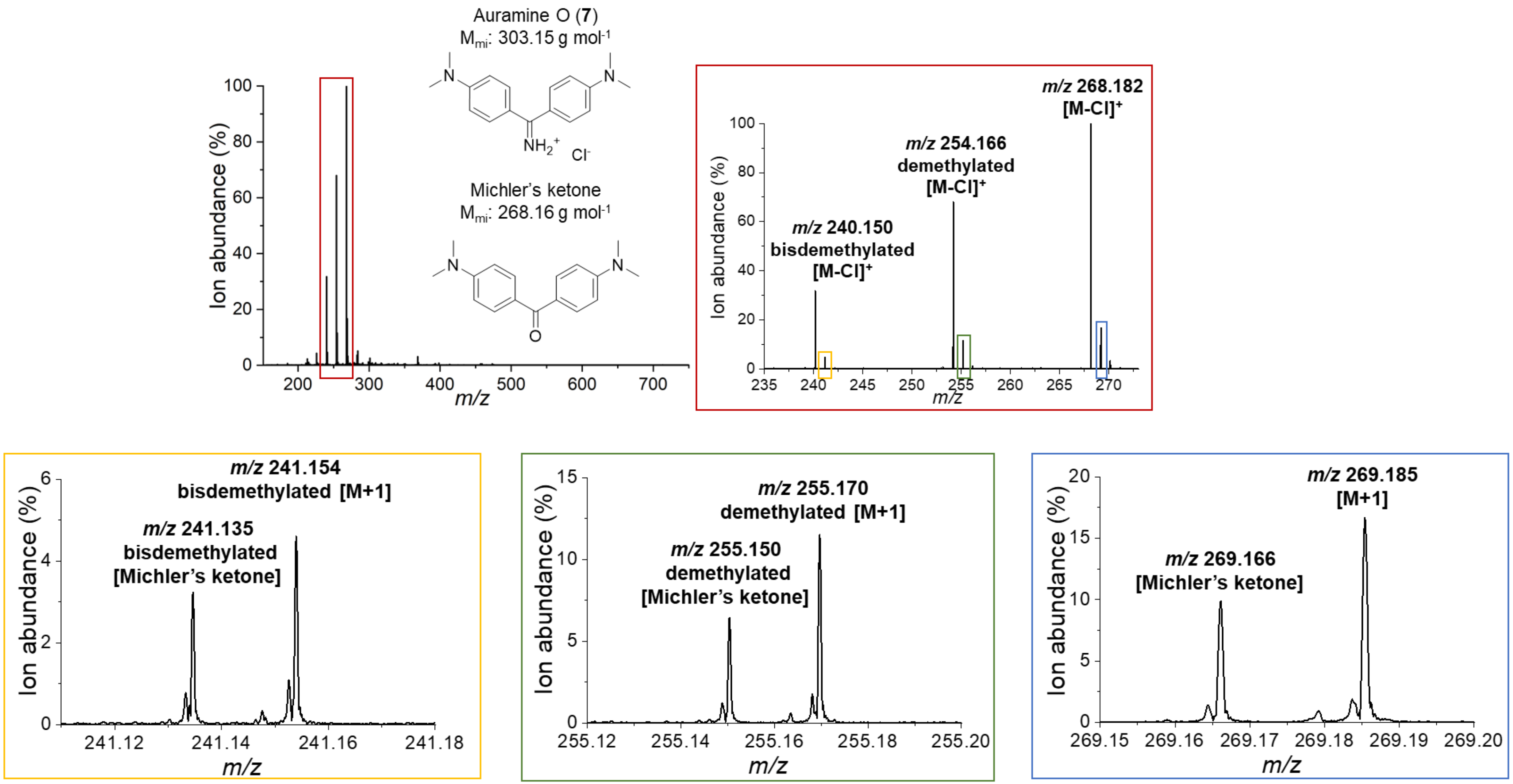

Figure S3. Top: DESI-MS spectrum of auramine O from Lehne's handbook (1893) with an expansion of the *m/z* region in the red box. Bottom: The presence of both the M+1 peak and likely Michler's ketone products for the parent ion and each demethylation product can be seen in the color coded expanded mass spectra.
